# Enhanced Proinflammatory Cytokine Production and Immunometabolic Impairment of NK Cells Exposed to *Mycobacterium tuberculosis* and Cigarette Smoke

**DOI:** 10.3389/fcimb.2021.799276

**Published:** 2022-01-05

**Authors:** Yafei Rao, Xiaoyan Gai, Yanqing Le, Jing Xiong, Yujia Liu, Xueyang Zhang, Jundong Wang, Wenli Cao, Yongchang Sun

**Affiliations:** ^1^ Department of Respiratory and Critical Care Medicine, Peking University Third Hospital, Beijing, China; ^2^ Department of Respiratory and Critical Care Medicine, Peking University International Hospital, Beijing, China; ^3^ Department of Infectious Diseases of Beijing Geriatric Hospital, Beijing, China

**Keywords:** chronic obstructive pulmonary disease (COPD), tuberculosis, cigarette smoking, lung tissue destruction, inflammation, immunometabolism, glycolysis, oxidative phosphorylation

## Abstract

**Aim:**

Smoker COPD patients with chest radiological signs of prior tuberculosis (TB) showed more severe lung damage, but the mechanisms remain unclear. Emerging evidence has implicated NK cells in the pathogenesis of both COPD and TB. The purpose of this study was to delineate the profile and cytokine production of NK-cell subpopulations and their immunometabolic changes after exposure to both cigarette smoke (CS) and *Mycobacterium tuberculosis*(MTB).

**Methods:**

We profiled NK-cell subpopulations in terms of percentage and cytokine production by flow cytometry in smoker patients with pulmonary TB (PTB). In an *in vitro* coexposure model, we investigated proinflammatory cytokine production, glycolytic influx, and oxidative phosphorylation of NK cells under CS extract (CSE) and PPD costimulation.

**Results:**

Peripheral blood NK cells in smoker patients with active PTB (CS+PTB group) showed altered proportion of subpopulations and excessive proinflammatory cytokine expressions. *In vitro*, CSE- and PPD-coexposed NK-92 cells displayed enhanced proinflammatory cytokine production, concurrent with decreased glycolytic influx and oxidative phosphorylation.

**Conclusion:**

Smoker patients with active PTB showed enhanced proinflammatory cytokine expression within altered NK cell subpopulations. CSE and PPD coexposure induced heightened cytokine production concurrent with impaired cell metabolism in NK cells. These novel data suggest a potential role of NK cells in the pathogenesis of lung injury in subjects with coexposure to CS and TB.

## Introduction

Chronic obstructive pulmonary disease (COPD) is a common chronic airway disease, which is characterized by persistent airway inflammation, lung tissue destruction (emphysema), and small airway fibrosis (Singh et al., 2019). The prevalence of COPD is increasing globally, particularly in low- and middle-income countries (LMICs) (Siddharthan et al., 2020). A cross-sectional study in mainland China found an overall prevalence of 8.6% of spirometry-defined COPD in adult populations (≥20 years old) (Wang et al., 2018). Cigarette smoke (CS) and biomass fuel exposure are the main risk factors for COPD (Wang et al., 2018), while in LIMCS with a high tuberculosis (TB) burden, TB is also an important risk factor (Singh et al., 2019).

Previous studies have revealed pulmonary *Mycobacterium tuberculosis* (Mtb) infection as a cause of airflow obstruction or spirometric restriction (Menezes et al., 2007; Lam et al., 2010; Amaral et al., 2015; Aggarwal et al., 2017). More recently, TB-associated COPD is gaining more attention as a unique phenotype, which is probably associated with persistent lung injury despite microbiological cure (Siddharthan et al., 2020). Our previous study showed that COPD patients (mostly smokers) with chest CT signs of prior tuberculosis had more severe emphysema and a higher prevalence of bronchiectasis, suggesting exaggerated lung injury in subjects with coexposure to CS and TB (Jin et al., 2018). However, the cellular and molecular mechanisms underlying lung injury in subjects with coexposure to CS and TB have been rarely studied.

Recent evidence has implicated NK cells in the pathogenesis of both COPD and TB (Travar et al., 2016; Liu et al., 2017; Osterburg et al., 2020; Rao et al., 2021). NK cells can be subdivided based on their CD56 expression: CD56^bright^ NK cells are generally associated with immunoregulatory properties and production of proinflammatory cytokines, while CD56^dim^ NK cells primarily exert cytotoxic functions (Newman and Riley, 2007; Osterburg et al., 2020; Rao et al., 2021). For COPD patients, the findings were varied in different studies on NK-cell receptors, cytotoxicity, and cytokine production (Prieto et al., 2001; Hodge et al., 2013; Tang et al., 2013; Yu et al., 2013; Finch et al., 2018). However, most studies have observed increased proinflammatory cytokine production and impaired cytotoxicity in smokers compared with healthy nonsmokers (Hughes et al., 1985; Hodge et al., 2013; Rao et al., 2021). Thus, we hypothesized that NK cells in CS-exposed subjects may present abnormally high proinflammatory cytokine production after Mtb infection, which may be involved in enhanced inflammation and tissue destruction such as those seen in TB-COPD.

Cell metabolism is considered to play an important role in determining the fate and function of immune cells. Different immune cells have different metabolic phenotypes adapted to cell needs (Pearce et al., 2013; Assmann et al., 2017; Alwarawrah et al., 2018; Poznanski and Ashkar, 2019). In the cell, glycolysis and oxidative phosphorylation are the two main pathways for energy production. Cells can shift between glycolysis and oxidative phosphorylation to adapt to alterations in their environment (Jose et al., 2011). Previous research indicates that glycolysis and oxidative phosphorylation are closely related to NK-cell cytotoxicity and IFN-gamma production (Donnelly et al., 2014; Keppel et al., 2015; Mah et al., 2017). Interestingly, studies observed altered metabolism in human macrophages from TB patients or peripheral blood mononuclear cells (PBMCs) from COPD patients (Cumming et al., 2018; Agarwal et al., 2019). Therefore, we asked whether CS and/or purified protein derivative tuberculin (PPD) exposure altered NK-cell function through effects on cell metabolism.

## Material and Methods

### Study Subjects

Eighty-two subjects were recruited in Peking University Third Hospital and Beijing Geriatric Hospital, including 21 healthy nonsmokers (HNS), 21 smokers without PTB (smoker group), 20 nonsmoker patients with active PTB (PTB group), and 20 active PTB patients with current cigarette smoking (CS+PTB group). As few women smoke, and therefore men account for the vast majority of smoker population in China, we only enrolled male subjects. The Ethics Committee of Peking University Third Hospital and Beijing Geriatric Hospital both approved this study (BJLNYY-ethic-2018-014). We obtained written informed consent from all subjects. Smoker subjects all had a smoking history of ≥10 pack-years. Active PTB was diagnosed according to the tuberculosis diagnostic criteria by Health Industry Standards of People’s Republic of China. All PTB and CS+PTB patients newly received the diagnosis of PTB without antituberculosis treatment when recruited. Subjects with other pulmonary diseases including asthma, interstitial lung diseases, bronchiectasis, malignant tumors, and autoimmune diseases were excluded.

### Flow Cytometry Analysis of PBMCs

PBMCs were obtained from HNS, smoker group, PTB group, and CS+PTB group. One milliliter peripheral blood was lysed with lysis buffer (BioLegend, San Diego, CA, USA) to remove red blood cells, and then viable cell count was obtained by trypan blue exclusion. After centrifugation for 10 min at 400×*g* at 4°C, cells were adjusted to 1 × 10^6^/ml and resuspended in 100 µl fluorescence-activated cell sorter (FACS) buffer (PBS supplemented with 0.5% bovine serum albumin and 2 mM EDTA). Fc blocker (BioLegend) was then added for removing nonspecific staining according to the manufacturers’ instruction. Cells were then stained with the following monoclonal antibodies on ice for 30 min protected from light for detecting membrane markers: Percp anti-human CD3(UCHT1), PE anti-human CD56(MEM-188), and BV510 anti-human CD16(3G8). After that, Zombie Green™ fixable viability and Zombie Aqua™ fixable viability dye were added for removing dead cells. For intracellular marker staining, cells were fixed with 4% paraformaldehyde (PFA) fixation for 10 min on ice and then permeabilized with fixation and permeabilization solution (BD) for 20 min and stained with following monoclonal antibodies on ice for 1 h: APC anti-human granzyme B (QA18A28), BV421 anti-human IFN-gamma (4S.B3), and PE-cy7 anti-human perforin (B-D48). The stained cells were measured through Beckman CytoFLEX S. Data were analyzed using FlowJo V10 software. Isotype controls and fluorescence minus one control were used to set gates. NK cell subpopulations per liter of peripheral blood were calculated according to the results of flow cytometry.

### CS Extracts Preparation

CS extracts (CSE) was prepared as described in our previous study (Xiong et al., 2021). In brief, five cigarettes were sucked through a 10-ml of RPMI-1640 medium at a constant velocity and then was sterilized *via* filtering through a 0.22-μm filter (Millipore, Burlington, MA, USA).

### Cell Culture and Treatment

The NK-92 cell line was purchased from Procell Biological Technology Co. Ltd. (Wuhan, China). PPD was a product of Xiangrui Biological Products Co., Ltd, Beijing, China. Cells were incubated with MEMα supplemented with 0.2 mM inositol, 0.1 mM β-mercaptoethanol+0.02 mM folic acid, 100 U/ml recombinant IL-2, 12.5% horse serum, 12.5% fetal calf serum, 1% penicillin and streptomycin under 37°C, and 5% CO_2_. NK-92 cells were stimulated with medium only (blank control), 1% CSE only (CSE group), 100 ng/ml PPD only (PPD group), and 1% CSE plus 100 ng/ml PPD (CSE- and PPD-coexposed group) for another 24 h. Cells were then harvested for the following experiments.

### Cell Viability Assay

Cell viability was detected by cell counting Kit-8 according to the manufacturer’s instructions. Briefly, NK-92 cells were seeded into 96-well plates at a density of 100,000 cells/well, then stimulated with 1% CSE and 100 ng/ml PPD, and finally incubated with CCK8 reagent for another 2 h. Absorbance was read in a spectrophotometer at a wavelength of 450 nm.

### Quantitative Real-Time Reverse Transcription PCR (qRT-PCR)

NK-92 cells were stimulated with 1% CSE and 100 ng/ml PPD for another 24 h. Subsequently, NK-92 cells were harvested. Gene expression of IFN-gamma, perforin, and granzyme B were determined by qRT-PCR. In brief, RNA was firstly isolated by the TRIzol reagent (Thermo Fisher Scientific, Waltham, MA, USA). Concentration of RNA was then assessed and reverse transcribed into cDNA using PrimeScript RT Reagent Kit (Vazyme, Nanjing, China). Finally, qPCR reactions were carried out with SYBR Green qPCR Mix (Vazyme, Nanjing, China), in a 20-µl reaction system with an ABI ViiATM 7 System. The primer sequences were IFN-gamma: 5′-AGCTCTGCATCGTTTTGGGT-3′ (forward) and 5′-TCCGCTACATCTGAATGACCT-3′ (reverse); perforin: 5′-TAACCAGGGCCAAAGTCAGC-3′ (forward) and 5′-CATTGCTGGTGGGCTTAGGA-3′ (reverse); granzyme B: 5′-TCAAAGAACAGGAGCCGACC-3′ (forward) and 5′-CTCTCCAGCTGCAGTAGCAT-3′ (reverse); IFN-α: 5′-AAGTCAAGCTGCTCTGTGGG-3′ (forward) and 5′-TGGAGGACAGGGATGGTTTC-3′ (reverse); IFN-β: 5′-TCTAGCACTGGCTGGAATGAG-3′ (forward) and 5′-GTGACTGTACTCCTTGGCCTT-3′ (reverse); actin: 5′-CACTCTTCCAGCCTTCCTT-3′ (forward) and 5′-AATGCCAGGGTACATGGTGG-3′ (reverse). Gene expression was calculated relative to that of actin in triplicates.

### Targeted Metabolomics Based on Multireaction Monitoring Technology

NK-92 cells were added with 1 ml methanol acetonitrile aqueous solution (2:2:1, V/V), vortexed for 60 s, then sonicated for 30 min at low temperature twice, followed by placing at −20°C for 1 h to precipitate protein, then centrifuged at 14,000 rcf 4°C for 20 min, and finally, the supernatants were freeze-dried and stored at −80°C. The samples were separated by Agilent 1290 infinity LC ultra-performance liquid chromatography system. Mobile phase: liquid A: 10 mm ammonium acetate solution and liquid B: acetonitrile. The sample was put in a 4°C autosampler with a column temperature of 45°C and a flow rate of 300°C µl/min, in sample size at 2 µl. The gradient of liquid phase was as follows: 0–18 min, liquid B changed linearly from 90% to 40%; 18–18.1 min, liquid B changed linearly from 40% to 90%; and 18.1–23 min, liquid B was maintained at 90%. A QC sample is set for a certain number of experimental samples at each interval in the sample queue, for the detection and evaluation of the stability and repeatability of the system. The standard mixture of energy metabolism substances was set up in the sample queue for the correction of chromatographic retention time. 5500 QTRAP mass spectrometer (AB SCIEX, Redwood City, CA, USA) was used for mass spectrometry analysis in negative ion mode. The source conditions of 5500 QTRAP ESI were as follows: source temperature 450°C, ion source gas 1 (Gas1): 45, ion source gas 2 (Gas2): 45, curtain gas (CUR): 30, Ion Sapary Voltage Floating (ISVF)–4,500 V; MRM mode was used to detect the ion pair. The peak area and retention time were extracted by Multiquant software. The retention time was corrected by the standard substance of energy metabolism substance; finally, the metabolites were identified.

### Extracellular Acidification Rate and Oxygen Consumption Rate Detection

Glycolytic flux and mitochondrial oxidative phosphorylation were detected by Extracellular Acidification Rate (ECAR) and oxygen consumption rate (OCR) with the XF96 Extracellular Flux Analyzer (Seahorse Bioscience, North Billerica, MA, USA). NK-92 cells were stimulated with 1% CSE and 100 ng/ml PPD for another 24 h. Sensor cartridges were treated with XF calibrant (200 μl/well) for 24 h at 37°C in a humidified incubator without CO_2_. Subsequently, 8 × 10^4^ NK-92 cells/well were adhered to a special 96-well plate using Cell-Tak as previously described (Corning^®^ Cell-Tak™ Cell and Tissue Adhesive, Corning, NY, USA) (Russell et al., 2019). Subsequently, cells were washed twice with the Seahorse XF stress test assay medium. Finally, the volume was controlled at 175 μl/well. The sequential injections in ECAR detection were glucose (10 mM), oligomycin (2 μM), and 2-deoxy-d-glucose (50 mM) while in OCR detection were oligomycin (1 μM), FCCP (0.125 μM), and rotenone/antimycin A (1 μM). Data were normalized to total protein content with a BCA assay.

### Glucose Consumption and Lactate Production Analysis

Glucose consumption and lactate production in supernatants were measured using a glucose assay kit (Solarbio, Beijing, China) and lactate assay kit (Solarbio, China), respectively. In brief, NK-92 cells were stimulated with 1% CSE and 100 ng/ml PPD for another 24 h. The supernatants were then harvested for the detection of glucose consumption and lactate production according to the manufacturer’s instructions. The absorbance was read in a spectrophotometer at a wavelength of 505 and 450 nm, respectively.

### Flow Cytometry Analysis of NK-92 Cells

For mitochondrial assays, NK-92 cells were stimulated with 1% CSE and 100 ng/ml PPD for another 24 h. NK-92 cells were then incubated with 50 nM Mito Tracker Green (MTG) (Beyotime Biotechnology Co., Ltd, Shanghai, China) and 12.5 nM Mito Tracker Deep Red (MTDR) (Invitrogen, Life Technologies, Carlsbad, CA, USA) in PBS for 30 min at 37°C prior to staining. The stained cells were measured through Beckman CytoFLEX S at 488/525 nm and 644/655 nm, respectively. Data were analyzed using FlowJo V10 software.

### Statistical Analysis

All data were shown as mean ± standard deviation (SD). Statistical analysis was processed with GraphPad Prism 7. One-way analysis of variance (ANOVA) with Bonferroni or Dunnett’s T3 posttest analysis was performed for comparisons between multiple groups when applicable. *p*-values less than 0.05 were considered statistically significant. Experiment data represent at least three independent experiments.

## Results

### Demographic Characteristics of Study Population

The study recruited 21 HNS, 21 smokers, 20 nonsmoker PTB patients, and 20 smoker PTB patients, all male, with a comparable age. **Table 1** shows the demographic and clinical characteristics of these subjects.

**Table 1:** Demographic characteristics of study population.

	HNS	Smoker	PTB	CS+PTB	*p*-value
Subjects (all males)	21	21	20	20	
Age (years)	49.39 ± 9.472	54.41 ± 6.167	47.9 ± 12.32	51.75 ± 12.72	NS
Smoking index (pack-year)	0	22.34 ± 9.92^*^	0	27.5 ± 12.54^#^	

HNS, healthy nonsmokers without PTB; Smoker, smokers without PTB; PTB, nonsmoker pulmonary tuberculosis patients; CS+PTB, pulmonary tuberculosis patients with current cigarette; NS, no significance. Smoking index = average number of cigarettes per day (pack) × number of years of smoking history (years). Mean ± SD.

^*^p < 0.05, smoker vs. HNS; ^#^p < 0.05, CS+PTB vs. HNS.

### Increased CD16^−^CD56^bright^ and CD16^+^CD56^dim^ NK-Cell Subpopulations in CS+PTB Patients

**Figure 1:**
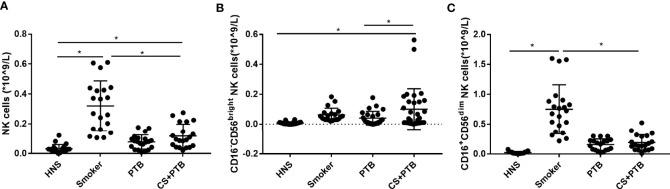
Increased CD16^−^CD56^bright^ and CD16^+^CD56^dim^ NK-cell subpopulation in CS+PTB patients. **(A)** Number of NK cells in HNS, smoker, PTB patients, and CS+PTB patients. **(B)** Number of CD16^−^CD56^bright^ NK-cell subpopulation in HNS, smoker, PTB patients, and CS+PTB patients. **(C)** Number of CD16^+^CD56^dim^ NK-cell subpopulation in HNS, smoker, PTB patients, and CS+PTB patients. One-way analysis of variance (ANOVA) with Bonferroni or Dunnett’s T3 posttest analysis was performed for comparisons between multiple groups. ^*^ *p* < 0.05.

In this study, we observed increased circulating NK cells and the CD16^−^CD56^bright^ NK-cell subpopulation in CS+PTB group as compared with the control group, but no difference was found in the CD16^+^CD56^dim^ NK-cell subpopulation (**Figures 1A–C**). The frequency of CD16^−^CD56^bright^ NK cells was higher in CS+ PTB group as compared with the HNS and PTB groups, suggesting that CS exposure drove the increase in the CD16^−^CD56^bright^ NK-cell subpopulation (**Figure 1B**).

### Increased Proinflammatory Cytokine Production in the CD16^-^CD56^bright^ and CD16^+^CD56^dim^ NK-Cell Subpopulations in CS+PTB Patients

**Figure 2:**
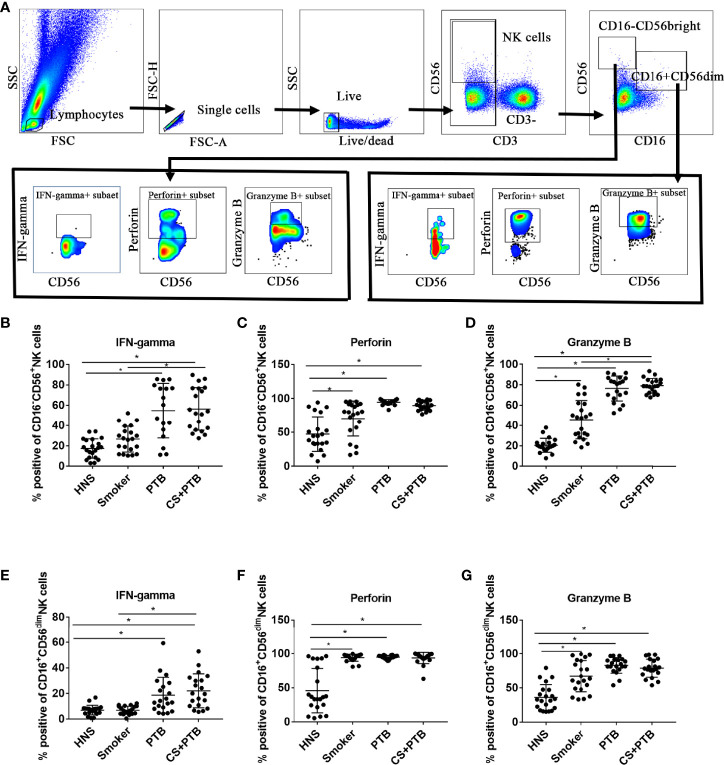
Increased proinflammatory cytokine production in both CD16^-^CD56^bright^ and CD16^+^CD56^dim^ NK-cell subpopulations in CS+PTB patients. **(A)** Flow cytometry gating strategy for identification of peripherial blood NK cell and cytokine production. **(B**–**D)** IFN-gamma^+^, perforin^+^, and granzyme B^+^ NK cell within CD16^−^CD56^bright^ NK-cell subpopulation were detected by flow cytometry. **(E**–**G)** IFN-gamma^+^, perforin^+^, and granzyme B^+^ NK cell within CD16^+^CD56^dim^ NK-cell subpopulation was detected by flow cytometry. One-way analysis of variance (ANOVA) with Bonferroni or Dunnett’s T3 posttest analysis was performed for comparisons between multiple groups. ^*^ *p* < 0.05.

Subsequently, we detected cytokine production in CD16^-^CD56^bright^ and CD16^+^CD56^dim^ NK cell subpopulations. **Figure 2A** shows the flow cytometry gating strategy for identification of peripherial blood NK cells and cytokines production. We observed that the percentages of IFN-gamma^+^ CD16^−^CD56^bright^ NK cells in the CS+PTB group and the PTB group were both higher as compared with the HNS group and the smoker group, while the latter two showed no differences (**Figure 2B**). Similar result was observed in the CD16^+^CD56^dim^ NK-cell population (**Figure 2E**). These data suggest that the increase in the percentage of IFN-gamma^+^ cells within both the CD16^−^CD56^bright^ and CD16^+^CD56^dim^ NK-cell subpopulations was mainly driven by Mtb infection. Moreover, we detected the production of perforin and granzyme B, two major molecules involved in cytotoxicity, in CD16^−^CD56^bright^ and CD16^+^CD56^dim^ NK-cell subpopulations (Esin and Batoni, 2015; Rao et al., 2021). For perforin, we found that the percentages of perforin^+^ cells within both CD16^−^CD56^bright^ and CD16^+^CD56^dim^ NK-cell subpopulations were higher in the smoker, PTB, and CS+PTB groups as compared with the HNS group, while the former 3 groups showed no differences (**Figures 2C, F** **)**, suggesting that both CS exposure alone and Mtb infection alone could drive overexpression of perforin in NK cells, with no observable synergism between the two. Similar patterns of granzyme B^+^ expression was also observed in these two subpopulations when compared among the four groups (**Figures 2D, G** **)**.

### CSE and PPD Coexposure Induced Proinflammatory Cytokine Production in NK-92 Cells

**Figure 3:**
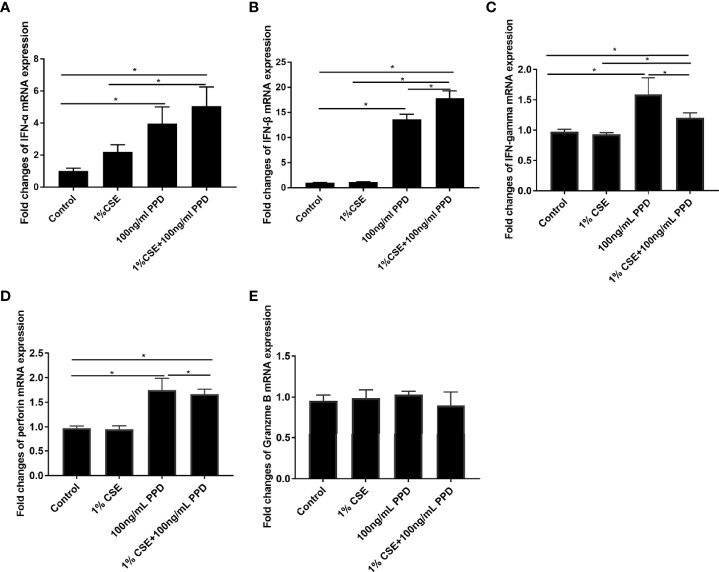
CSE and PPD coexposure induced proinflammatory cytokine production in NK-92 cells. NK-92 cells were stimulated with 1% CSE and/or 100 ng/ml PPD accordingly for another 24 h The specific grouping is as follows: control (control group), 1% CSE (CSE group), 100 ng/ml PPD (PPD group), 1% CSE+100 ng/ml PPD (CSE- and PPD-coexposed group). mRNA expressions of IFN-α **(A)**, IFN-β **(B)**, IFN-gamma **(C)**, perforin **(D)**, and granzyme B **(E)** were assessed by qRT-PCR. *n* ≥ 3 independent experiments. One-way analysis of variance (ANOVA) with Bonferroni or Dunnett’s T3 posttest analysis was performed for comparisons between multiple groups. ^*^ *p* < 0.05.

In order to confirm the findings of enhanced proinflammatory cytokine production by NK cells in PTB patients with smoking, we performed *in vitro* experiments on NK-92 cells. To determine the optimal doses of CSE and PPD, we firstly assessed the cellular toxicity of CSE at concentrations ranging from 1% to 5% and PPD ranging from 10 to 100 ng/ml. After stimulation for 24 h, cell viability was assessed (**Supplementary Figure S1**). Accordingly, 1% CSE and 100 ng/ml PPD coexposure for 24 h was applied in following experiments. We observed that the mRNA levels of IFN-α, IFN-β, IFN-gamma, and perforin in the CSE- and PPD-coexposed (1% CSE+100 ng/ml PPD) group and PPD group were higher as compared with the control group (**Figures 3A–D**), suggesting that the increased expressions of these cytokines by NK-92 cells were mainly driven by PPD treatment. As to granzyme B mRNA expression in NK-92 cells, no difference was observed among the groups (**Figure 3E**).

### Decreased Glycolytic Flux in NK-92 Cells Upon CSE and PPD Exposures

**Figure 4:**
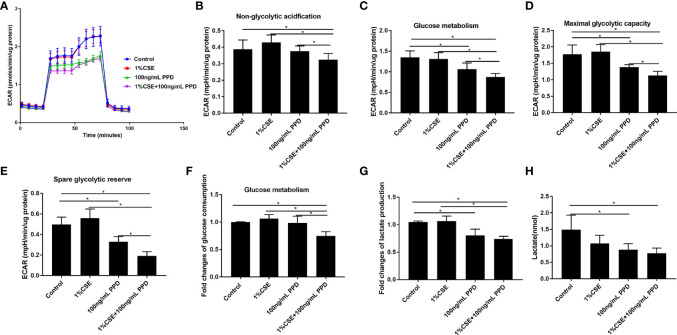
Decreased glycolytic flux in NK-92 cells upon CSE and PPD exposure. NK-92 cells were stimulated with 1% CSE and/or 100 ng/ml PPD accordingly for another 24 h The specific grouping is as follows: control (control group), 1% CSE (CSE group), 100 ng/mL PPD (PPD group), 1% CSE+100 ng/mL PPD (CSE- and PPD-coexposed group). NK-92 cells were then plated onto Seahorse Bioscience plates. **(A)** Representative glycolytic stress tests were performed with sequential addition of glucose (10 mM), oligomycin (2 μM), and 2-deoxy-d-glucose (50 mM). **(B**–**E)** Indices of glycolytic pathway (nonglycolytic acidification, glucose metabolism, maximal glycolytic capacity, and spare glycolytic reserve) of CSE- and PPD-coexposed NK-92 cells. **(F, G)** Glucose consumption and lactate production were detected. **(H)** Changes in major metabolic changes related to cellular energetic pathways (glycolysis) were detected by LC-MS/MS. *n* ≥ 3 independent experiments. One-way analysis of variance (ANOVA) with Bonferroni or Dunnett’s T3 posttest analysis was performed for comparisons between multiple groups. ^*^ *p* < 0.05.

To evaluate the functional immunometabolism in CSE- and PPD-coexposed NK-92 cells, we performed real-time measurement of metabolic rate with Seahorse analyzer. The Seahorse XFp glycolysis stress test was carried out to assess glycolysis by the continuous adjunction of glucose, oligomycin, and 2-DG (**Figure 4A**).

Nonglycolytic acidification and glucose metabolism were analyzed before and after addition of glucose, respectively. NK-92 cells in CSE- and PPD-coexposed group demonstrated decreased nonglycolytic acidification and glucose metabolism as compared with the control group, CSE group, and PPD group (**Figures 4B, C** **)**, suggesting that CSE and PPD coexposure decreased nonglycolytic acidification and glucose metabolism, with a synergistic effect. In addition, NK-92 cells in the CSE- and PPD-coexposed group displayed decreased maximal glycolytic capacity and spare glycolytic reserve as compared with other groups (**Figures 4D, E** **)**.

We further measured the glucose consumption (raw materials for glycolysis) and lactate production (the end product of the glycolytic pathway) in retained supernatants. NK-92 cells in CSE- and PPD-coexposed group displayed decreased glucose consumption as compared with the control group, CSE group, and PPD group, while no differences were observed between the latter three groups (**Figure 4F**), suggesting that CSE and PPD acted synergistically to decrease glycolysis. We also found that NK-92 cells in the CSE- and PPD-coexposed group and in the PPD group, but not in the CSE group, showed decreased lactate production (**Figure 4G**), indicating that PPD but not CSE decreased cell glycolysis. In concordance with extracellular flux, glucose consumption, and secreted lactate measurements, liquid chromatography-tandem mass spectrometry (LC-MS/MS)-based profiling on NK-92 cells revealed decreased glycolysis, as manifested by decreased lactate production, in CSE- and PPD-coexposed group and PPD group (**Figure 4H**), indicating that PPD exposure exerted a major effect on decreasing glycolytic flux.

### Decreased Oxidative Phosphorylation in NK-92 Cells Upon CSE and PPD Exposure

**Figure 5:**
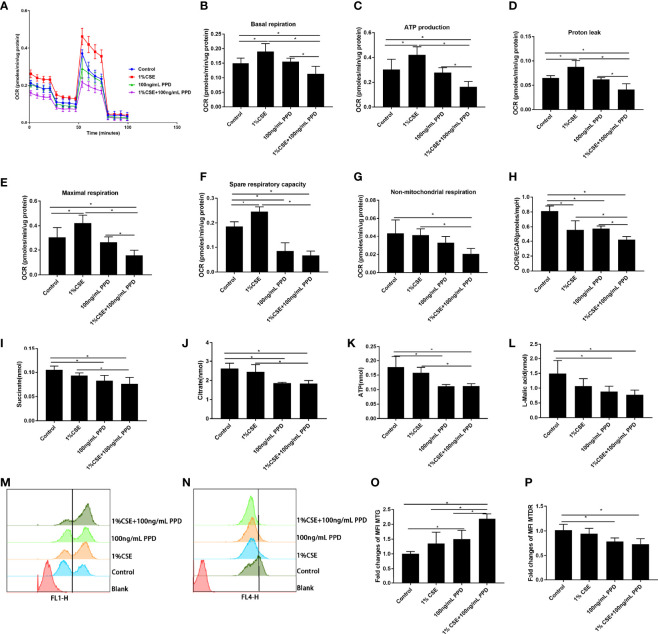
Decreased oxidative phosphorylation in NK-92 cells upon CSE and PPD exposure. NK-92 cells were stimulated with 1% CSE and/or 100 ng/ml PPD accordingly for another 24 h The specific grouping is as follows: control (control group), 1% CSE (CSE group), 100 ng/ml PPD (PPD group), 1% CSE+100 ng/ml PPD (CSE- and PPD-coexposed group). NK-92 cells were then plated onto Seahorse Biosciences plates. **(A)** Representative mitochondrial stress tests were conducted with sequential addition of oligomycin (1 μM), FCCP (0.125 μM), rotenone/antimycin A (1 μM). **(B**–**H)** Indices of mitochondrial respiratory function (basal respiration, ATP production, proton leak, maximal respiration, spare respiration capacity, and nonmitochondrial respiration and OCR/ECAR ratio) of CSE- and PPD-coexposed NK-92 cells. **(I**–**K)** Changes in major metabolic intermediate (succinate, citrate, ATP, l-malic acid) changes related to cellular energetic pathways (TCA cycle) were detected by LC-MS/MS. **(L**–**O)** Mitochondrial mass and mitochondrial membrane potential were detected by flow cytometry. *n* ≥ 3 independent experiments. One-way analysis of variance (ANOVA) with Bonferroni or Dunnett’s T3 posttest analysis was performed for comparisons between multiple groups. ^*^ *p* < 0.05.

For the detection of metabolic responses of NK-92 cells to CSE and PPD, we performed real-time measurement of metabolic rate with Seahorse analyzer. The Seahorse XFp Mito stress test was carried out to assess oxidative phosphorylation by the continuous addition of oligomycin, carbonyl cyanide-4-(trifluoromethoxy) phenylhydrazone (FCCP), and rotenone/antimycin A (**Figure 5A**).

NK-92 cells in CSE- and PPD-coexposed group demonstrated least basal respiration, proton leak, ATP production, and maximal respiration as compared with control group, CSE group, and PPD group (**Figures 5B–E**), suggesting that CSE and PPD acted synergistically to decrease basal respiration, proton leak, ATP production, and maximal respiration. Additionally, NK-92 cells in CSE- and PPD-coexposed group demonstrated decreased spare respiratory reserve and nonmitochondrial respiration as compared with control group and CSE group (**Figures 5F, G**), indicating that PPD exposure exerted a major effect on decreasing spare respiratory reserve and nonmitochondrial respiration. Moreover, NK-92 cells in CSE- and PPD-coexposed group showed decreased OCR/ECAR ratio as compared with the control group, suggesting a shift away from oxidative phosphorylation toward glycolysis in the cells (**Figure 5H**).

For further confirmation, we performed LC-MS/MS-based profiling on NK-92 cells for the detection of intermediate accumulation in TCA cycle. NK-92 cells in CSE- and PPD-coexposed group showed lower TCA cycle intermediate accumulation (succinate, citrate, ATP, and l-malic acid) as compared with the control group and CSE group (**Figures 5I–L**), suggesting that the decrease in intermediate accumulation in TCA cycle was mainly driven by PPD exposure.

To investigate whether the decline in oxidative phosphorylation observed in NK-92 cells exposed to CSE and PPD could be due to an accumulation of dysfunctional mitochondria, we assessed MM and MMP in NK-92 cells. Flow cytometry gating strategy for determination of mitochondrial mass and mitochondrial membrane potential of NK-92 cells (**Supplementary Figure S2**). CSE- and PPD-coexposed NK-92 cells displayed higher intensity of MM as compared with the control group, CSE group, and PPD group (**Figures 5M, O** **)**, suggesting that the increase in the percentage of MM was driven by both CSE and PPD. Furthermore, CSE- and PPD-coexposed NK-92 cells showed lower intensity of MTDR as compared with the control group, so did the PPD group (**Figures 5N, P** **)**, indicating that PPD exposure exerted a major effect on decreasing MTDR.

## Discussion

In the present study, for the first time to our knowledge, we delineated the profiles of NK cell subpopulations in terms of percentage and cytokine production in PTB patients concurrent with significant CS exposure. We observed similar frequency of circulating NK cells (% of total lymphocytes) but different frequency of its subpopulations among HNS, smokers, PTB patients, and smoker PTB(CS+PTB) patients. The frequency of CD16^−^CD56^bright^ NK cells was higher in the CS+PTB group as compared with the HNS, smoker, and PTB groups, while the latter 3 groups showed no differences, suggesting that Mtb infection combined with CS exposure drove the increase in the CD16^−^CD56^bright^ NK cell subpopulation. Interestingly, the CD16^+^CD56^dim^ NK cell subpopulation was also increased in both the smoker group and the CS+PTB group, as compared with the HNS and the PTB groups, suggesting that CS exposure alone drove the increase in the CD16^+^CD56^dim^ NK cell population. These data indicate that Mtb infection and CS exposure may act synergistically or complementarily in inducing altered NK cell homeostasis.

Inflammation is a protective response, which protects and repairs tissues under infection, injury, or stress (Medzhitov, 2008). Under certain circumstances, inflammation persists, giving rise to chronic inflammation and tissue damage (Furman et al., 2019), such as in COPD and PTB. In this study, we detected cytokine production in CD16^−^CD56^bright^ and CD16^+^CD56^dim^ NK cell subpopulations. We observed that the percentages of IFN-gamma^+^ CD16^−^CD56^bright^ NK cells in the CS+PTB group and the PTB group were both higher as compared with the HNS group and the smoker group, while the latter two showed no differences. Similar result was observed in the CD16^+^CD56^dim^ NK cell population. These data suggest that the increase in the percentage of IFN-gamma^+^ cells within both the CD16^−^CD56^bright^ and CD16^+^CD56^dim^ NK-cell subpopulations was mainly driven by Mtb infection. Moreover, we detected the production of perforin and granzyme B, two major molecules involved in cytotoxicity, in CD16^−^CD56^bright^ and CD16^+^CD56^dim^ NK cell subpopulations (Esin and Batoni, 2015; Rao et al., 2021). The results suggest that both CS exposure alone and Mtb infection alone could drive overexpression of perforin in NK cells, with no observable synergism between the two. Similar patterns of granzyme B^+^ expression was also observed in these two subpopulations. Production of perforin and granzyme B^+^ by NK cells is critical for innate immunity against Mtb infection, but overexpression of these molecules may elicit injury to host tissues, such as those seen in COPD or PTB. To recapitulate the changes seen in our patients, we examined NK cells exposed to both CSE and PPD in an *in vitro* model. We found that the patterns of IFN-gamma and perforin expression were similar to findings in human subjects, while no difference was observed for granzyme B expression. In the cell, glycolysis and oxidative phosphorylation are the two main pathways for energy production (Jose et al., 2011). Cell metabolism plays an important role in determining the fate of immune cells and impacts the inflammatory response (Pearce et al., 2013; Assmann et al., 2017; Alwarawrah et al., 2018; Poznanski and Ashkar, 2019). Previous studies suggested altered metabolism in human macrophages of PTB patients and PBMCs of COPD patients (Cumming et al., 2018; Agarwal et al., 2019). Therefore, we explored the glycolysis and oxidative phosphorylation of NK-92 cells exposed to CSE and/or PPD. NK-92 cells in CSE- and PPD-coexposed group displayed decreased glycolytic flux including glucose metabolism, nonglycolytic acidification, maximal glycolytic capacity, spare glycolytic reserve, and impaired oxidative phosphorylation including ATP production, maximal respiration, maximal respiration, and proton leak as compared with the control group, CSE group, and PPD group. PPD exposure alone led to reduced glycolytic flux including glucose metabolism, nonglycolytic acidification, maximal glycolytic capacity, and spare glycolytic reserve and impaired oxidative phosphorylation as manifested by reduced spare respiratory capacity. While NK-92 cells in the CSE group exhibited no changes in glycolytic flux but increased oxidative phosphorylation as compared with the control group. These data suggest that CSE and PPD could act synergistically or complementarily to induce NK cells to undergo metabolic programming.

As has been illustrated above, NK-92 cells in CSE- and PPD-coexposed group displayed increased proinflammatory cytokine production (IFN-α, IFN-β, IFN-gamma, and perforin), concurrent with decreased glycolytic flux and oxidative phosphorylation, which was in line with a previous study showing that GAPDH expression (glycolytic enzyme) inversely correlated with IFN-gamma production in CD4 T cells *in vivo* after infection with *Listeria monocytogenes (* Chang et al., 2013). These findings could be explained as follows. Firstly, glycolytic enzymes could bind proinflammatory genes by binding to the AU-rich region in the 3′-UTR of cytokine mRNA, thus inhibiting their translation. This was determined by whether or not the glycolytic enzyme was occupied by its metabolic function or by its expression level in cells (Chang et al., 2013). In our study, although both glycolysis and oxidative phosphorylation decreased in CSE- and PPD-coexposed NK-92 cells, the OCR/ECAR ratio still increased, indicating that the cells still showed a glycolytic phenotype, and thus the glycolytic enzyme was occupied by its metabolic function, leading to less binding of glycolytic enzyme with proinflammatory genes, and eventually leading to increased proinflammatory cytokine production. Secondly, glycolytic enzyme function is heavily affected by the redox state of a cell, and thess redox-mediated alterations can change its function (Colell et al., 2007; Tristan et al., 2011). Thirdly, during the process of metabolic reprogramming, cells may accumulate a variety of metabolites which can modulate the inflammatory status through different mechanisms. However, the final inflammatory outcome was determined by the ratio between metabolites (Soto-Heredero et al., 2020). It is likely that many of these processes contribute to enhanced proinflammatory cytokine production in our experimental model.

Our study has several limitations. Firstly, for clinical feasibility, we enrolled active PTB patients with current smoking but not COPD patients with active PTB. Therefore, our findings are not directly relevant to COPD but only suggest potential mechanisms needing further investigation. Secondly, for *in vitro* experiments, we used PPD instead of live *Mycobacterium tuberculosis* or *Bacillus* Calmette-Guérin, although PPD is also commonly used in related studies (Hussain et al., 2000; Marín et al., 2017). Thirdly, for the human study, because bronchoscopy was not clinically indicated for the patients, we investigated only blood NK cells but not NK cells in bronchoalveolar lavage (BAL) which could be more relevant to diseases of the lung.

In conclusion, we found higher percentages of CD16^−^CD56^bright^ and CD16^+^CD56^dim^ NK-cell subpopulations in smoker patients with active PTB, with increased proportions of IFN-gamma^+^, perforin^+^, and granzyme B^+^ cells. Enhanced proinflammatory cytokine expression was partly recapitulated in an *in vitro* model of NK-92 cells coexposed to CSE and PPD, and this was concurrent with impaired cell metabolism. These novel data highlight a potential role of NK cells in persistent inflammation and exaggerated lung injury seen in tuberculosis-associated COPD and suggest related mechanisms for further investigation.

## Data Availability Statement

The original contributions presented in the study are included in the article/**Supplementary Material**. Further inquiries can be directed to the corresponding authors.

## Ethics Statement

The studies involving human participants were reviewed and approved by The Ethics Committee of Peking University Third Hospital and Beijing Geriatric Hospital. The patients/participants provided their written informed consent to participate in this study.

## Author Contributions

YR, WC, and YS designed the experiments. YR, XG, YaL, JX, YuL, XZ, and JW recruited the patients, collected samples, and analyzed the clinical data. YR and YS wrote the paper. All authors contributed to the article and approved the submitted version.

## Funding

This study was financially supported by the National Natural Science Foundation of China (81970041, 81770040) and Natural Science Foundation of Beijing Municipality (7192224).

## Conflict of Interest

The authors declare that the research was conducted in the absence of any commercial or financial relationships that could be construed as a potential conflict of interest.

## Publisher’s Note

All claims expressed in this article are solely those of the authors and do not necessarily represent those of their affiliated organizations, or those of the publisher, the editors and the reviewers. Any product that may be evaluated in this article, or claim that may be made by its manufacturer, is not guaranteed or endorsed by the publisher.
