# Supplementary material for: Enhanced Proinflammatory Cytokine Production and Immunometabolic Impairment of NK Cells Exposed to Mycobacterium tuberculosis and Cigarette Smoke
Source: Front Cell Infect Microbiol. 2022 Jan 5;11:799276. doi: 10.3389/fcimb.2021.799276 (PMC8766853; doi:10.3389/fcimb.2021.799276)
Supplement: Supplementary file 1 [file DataSheet_1.docx]

Supplementary Materials


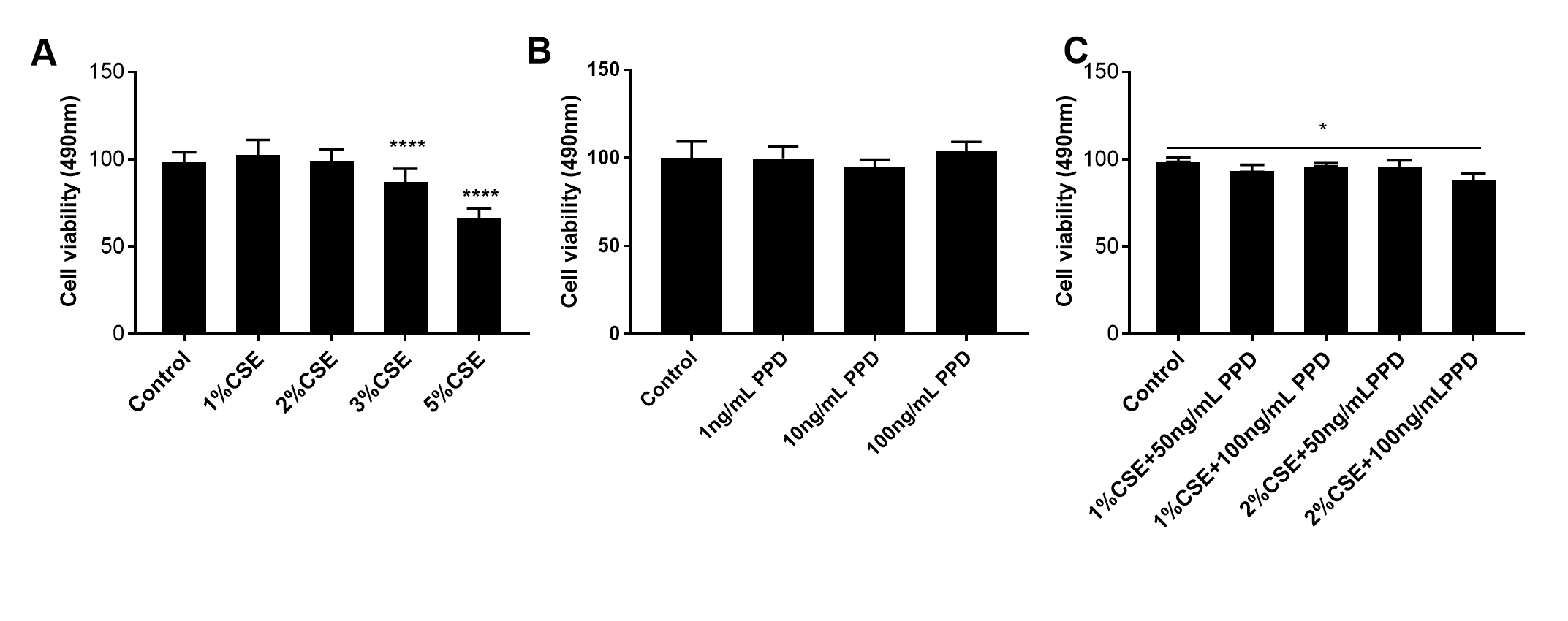


**Supplementary Figure 1.** Various concentrations of CSE and PPD were used to stimulate NK-92 cells for 24 h, and then CCK8 assays were used to measure the cell viability. *P* < 0.05. N =6.


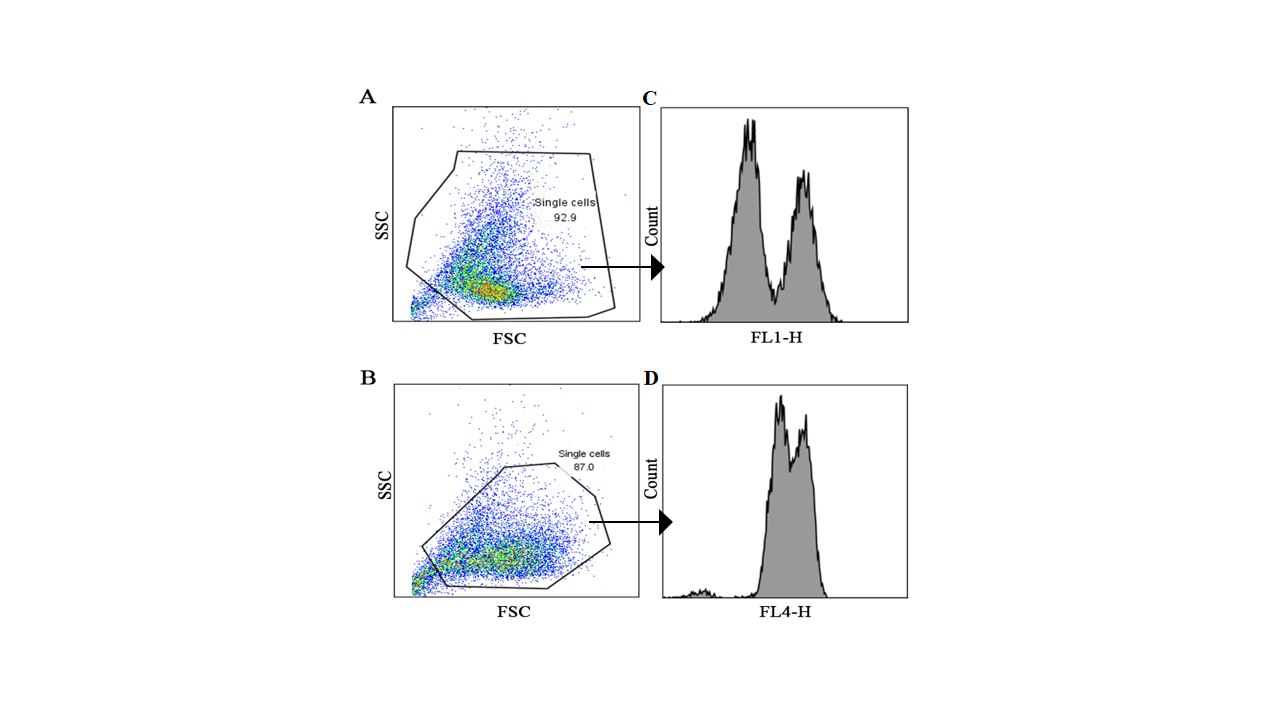


**Supplementary Figure 2.** Flow cytometry gating strategy for determination of mitochondrial mass and mitochondrial membrane potential of NK-92 cells. A,B. Single NK-92 cells (excluding debris (FSC^low^/SSC^low^) and doublets). C,D Signals in FL1-1(mitochondrial mass) and FL4-H (mitochondrial membrane potential)were collected.
